# Infection prevention behaviour among hospital nursing staff: Navigating in a complex and shifting work environment

**DOI:** 10.1177/17571774251322449

**Published:** 2025-02-20

**Authors:** Lisa Arvidsson, Maria Lindberg, Bernice Skytt

**Affiliations:** 1 Department of Caring Sciences, Faculty of Health and Occupational Studies, Gävle, Sweden; 2 Centre for Research and Development, Uppsala University/County Council of Gävleborg, Gävle, Sweden; 3 Department of Public Health and Caring Sciences, Uppsala University, Uppsala, Sweden

**Keywords:** Compliance, focus groups, infection prevention behaviours, qualitative research, working conditions

## Abstract

**Background:**

Healthcare-associated infections are a global concern and can be dependent on the infection prevention behaviours of nursing staff, which in turn can be influenced by working conditions. Qualitative studies are scarce, and a greater understanding of the relationship between working conditions and nursing staff behaviour is needed.

**Aim:**

The aim was to describe nursing staff’s experiences and reflections on working conditions and infection prevention behaviours.

**Methods:**

A qualitative study with semi-structured focus group interviews at four surgical units and two orthopaedic hospital units. Twenty-seven nursing staff (12 registered nurses and 15 assistant nurses) participated. Data was analysed using qualitative content analysis.

**Results:**

We generated one theme: Navigating in a complex and shifting context. The result indicates that working conditions are sometimes inadequate, which can hinder the nursing staff’s infection prevention behaviours. Even when working conditions seemed to be sufficient, hygiene routines could fail, since situations constantly arise in a hospital unit that are difficult to predict and regulate.

**Discussion:**

This study highlights the complexities faced by nursing staff in maintaining infection prevention behaviours within the dynamic hospital work environment. While nursing staff are professionally obliged to comply with hygiene routines, organisational support is essential for fostering sustainable working conditions. A multi-tiered approach is needed, from first-line managers to decision-makers, to promote a supportive environment that sustains safe practices.

## Background

Healthcare-associated infections (HCAIs) are the most frequently reported adverse event and a significant threat to patient safety worldwide (Haque et al., 2018; World Health Organization, 2011). While many infections are influenced by factors such as patient age, immunosuppression, invasive procedures, and length of hospital stay (World Health Organization, 2011), the infection prevention behaviours of healthcare professionals also play a crucial role (Loveday et al., 2014). When it comes to behaviours, non-compliance with hand hygiene is widely regarded as the most significant risk for organism transmission (Allegranzi and Pittet, 2009). However, a range of other situations and behaviours may also contribute to such transmission, for example, inappropriate use of gloves and protective clothing, uncleaned medical devices, and lack of surface disinfection (Loveday et al., 2014).

Human risk behaviour is influenced by working conditions, since behaviour is a causal part of a sequence of events affected by the environment (Rasmussen, 2003). Worldwide, nurses are reporting inadequate working conditions (Goodare, 2017). Working conditions cover aspects from psychological demands to physical workplace conditions (Eurofound and International Labour Organization, 2019). Previous research on working conditions and infection prevention behaviours has largely been quantitative, focussing mainly on the frequency of HCAI or compliance with hand hygiene. Several working conditions that affect the frequency of HCAI have been identified, such as bed occupancy, staffing levels, workload, use of pool or agency nurses, and availability of materials (Zingg et al., 2015). However, qualitative studies on this topic are limited. A recently published study investigated how nurses adapted their work environment and infection prevention behaviours during the COVID-19 pandemic, highlighting the influence of team dynamics, leadership support, and knowledge-sharing on infection prevention practices (De Vos et al., 2024) Moreover, research would benefit from focussing on various infection prevention behaviours, not just hand hygiene. Since registered nurses and assistant nurses (hereafter referred to as nursing staff) in hospital units are two professional groups that come in close contact with patients, it is appropriate to start by investigating their experiences and reflections. Investigating this qualitatively makes it possible to gain insight into the views of nursing staff on how best to prevent organism transmission. HCAIs are a global concern and need to be addressed in several ways. Gaining a greater understanding of the working conditions for healthcare professionals is one way to ensure that appropriate measures can be implemented to improve patient safety in hospital care.

### Aim

The aim was to describe nursing staff’s experiences and reflections on working conditions and infection prevention behaviours.

## Methods

### Design

A qualitative descriptive study was conducted between April and December 2023. The ‘Consolidated criteria for reporting qualitative research (COREQ): a 32-item checklist for interviews and focus groups’ was used to report the study design and findings (Tong et al., 2007).

### Setting and sample

A purposive selection of the setting was employed, drawing upon previous research conducted by the research team, where eight hospital units previously involved in a mixed methods study (Arvidsson et al., 2021) were invited to participate. Four surgical and two orthopaedic units from five Swedish hospitals accepted, while two orthopaedic units declined participation due to high workload. Although the focus groups were intended to consist of 6–8 participants, they ultimately ranged from 3 to 7. First-line managers assisted in scheduling interviews. All 27 participants (12 registered nurses and 15 assistant nurses; 26 female) completed data collection. Ages ranged from 19 to 64 years (M = 39.5), with 0.5–40 years of work experience (M = 11.5) and 0.5–25 years at their current unit (M = 4.8).

### Data collection

One face-to-face focus group interview was conducted per unit in a nearby room, lasting 46 to 63 minutes. Participants completed background questions before the interview. The first author (LA) moderated the interviews, with the last author (BS) assisting by taking notes. A semi-structured interview guide with open-ended questions, derived from two prior studies on working conditions and infection prevention (Arvidsson et al., 2021, 2023), guided the interviews. The areas covered interruptions, patient room arrangements, psychosocial work environment, structural empowerment, work engagement, and work-related stress. A pilot interview was conducted to refine the guide, resulting in minor adjustments. All interviews were audio recorded.

### Data analysis

The audio recordings were transcribed verbatim, with observations from the mind map notes incorporated into the transcripts (e.g. participant agreement or nodding). Thereafter, the interviews were analysed using qualitative content analysis (Graneheim et al., 2017).

Initially, transcripts were read repeatedly for an overall understanding, followed by thorough reading to identify meaning units relevant to the study’s aim. The meaning units were condensed, coded, and then compared based on differences and similarities, leading to the formation of categories. During this phase, a theme was generated to express the latent content in the categories. This process was characterised by a forward and backward movement between the whole and the parts of the text (Graneheim et al., 2017). The first author performed the analysis in a continuous dialogue with the research team.

## Results

The theme *Navigating in a complex and shifting context* was generated from the finding. The nursing staff reported that a variety of situations and factors influenced their infection prevention behaviours, ranging from physical work environment and workplace climate to workload and individual choices. Inadequate working conditions could lead to both conscious and unconscious deviations. But even when working conditions seem to be sufficient, compliance with hygiene routines is not always an easy task, as situations constantly arise in hospital units that are difficult to predict and regulate. The theme consists of four categories (described below), and the results are presented with quotations from the focus group interviews.

### The influence of co-workers and the workplace climate

The participants noted the psychosocial work environment, attitudes, and commitment as crucial for their infection prevention behaviours, and described compliance as a herd behaviour. They reported that instead of speaking up, they would sometimes go along with the behaviour of those around them (e.g. by simply handing over an extra apron), especially when they believed that criticism would not be well received. The participants highlighted the importance of quickly training new colleagues and bringing them into a close-knit group. If this is achieved, a feeling of security emerges, enabling an open discussion about hygiene issues. However, many units struggled with high staff turnover, which participants believed could affect the working climate and infection prevention behaviours. *I also think a lot about the climate in our department, that we have a lot of new staff training new staff. There are a lot of new staff where the routines in our department have not been established, but they still have to train the next person, so there are a lot of things that fall off that used to be set in stone* (RN2) (Interview 1).

The participants noted the importance of having a manager committed to hygiene issues and formal hygiene representatives, preferably with the inclusion of hygiene awareness events. They highlighted the importance of having good knowledge of infection prevention. However, the difference between general knowledge and true understanding was emphasised. *It's a lot like ‘this is what we should do’ but maybe not so much why we should do it, or what will happen if we don't do it* (RN1) (Interview 5).

### Interruptions raise the risk of deviations in hygiene routines but are also a necessary part of work progress

Participants described interruptions as a factor that negatively affected infection prevention behaviours due to a loss of focus. They reported that interruptions could be caused by a variety of situations. *Someone comes in from the emergency department* (RN1) *‘This one has very low blood pressure’* (AN5). *Yes, it is common to be interrupted because there is always something happening* (RN4) *Yes, it really does* (RN2) (Interview 4). However, at the same time, interruptions were described as a central part of the nursing work. For example, colleagues often need to share information, which was emphasised as crucial for patient safety. On the other hand, interruptions present a threat when they occur at inappropriate times. *It is difficult not to interrupt if you want to be able to communicate because you are always doing something, so somewhere in all those moments, you have to communicate, so you will be interrupted. But I sometimes feel that people maybe don’t have enough consideration for each other in deciding when it is appropriate to interrupt and when it isn’t* (RN2) (Interview 5). Participants stated that registered nurses are particularly exposed to interruptions, since they are contacted by various healthcare professionals regarding the patient’s care. The registered nurses reported that telephone interruptions were particularly common.

### Access to adequate premises, materials, and equipment is essential but no guarantee of compliance

According to the participants, physical conditions at the workplace, such as strategic placement of hand disinfection, gloves, and aprons, were critical for infection prevention behaviours. Access to these materials was generally described as good, but sometimes lacking in specific areas (such as toilets). *They are in such a terrible hurry when they go to the toilet, some of them. Instead of waiting for us to get dressed…* (AN2) *The fact we don't have it in the toilet, then we have to let go of them even though they are unsteady and go out and get them* (RN1). *Yes* (AN2). *Then we may choose unhygienic practices (RN1). Yes* (AN2) (Interview 1). The participants also emphasised the importance of everyone taking responsibility and replenishing materials (e.g. gloves) to avoid putting the colleague working the next shift in a bad position.

In some units, participants faced challenges with clothing routines due to shortages of clean work clothes. Other vital equipment included wastepaper baskets, linen hampers, toilets, and bedpan washers, preferably adjacent to each patient room. Despite access to necessary equipment, complex situations could complicate the work. Participants often wished for having an extra hand to manage tasks independently to prevent spread of infection. Furthermore, contaminated materials could sometimes end up in inappropriate places due to the absence of work surfaces or work disruptions, such as a busy bedpan washers. The participants reflected on the handling of used urinal bottles when the bedpan washer is not directly adjacent to the patient room. *I’ll probably go with the bottle right away* (RN4). *Not with the protective apron, we’re not allowed to go out in the hallway with that* (RN3). *I wear the apron* (AN5). *Yes, me too* (RN4). *But I dress without an apron but with gloves, because we can’t go out in the hallway with an apron* (RN3). *Yes, for the sluice room, if we have contaminated material* (AN5). *I take off the apron. Because if you have something on your apron, you bring it out into the hallway,* *but I don’t know if that’s right* (RN3). *You don’t want to say, ‘well, I accidentally tripped and then I got pee all over myself’* (RN4) *Several laugh.* *Then you have to put the urinal bottle down and then you put on the apron and new gloves* (AN5). *No, I hold the urinal bottle, then I’ll take it off* (demonstrates with hands) (RN3) (Interview 2). Participants described how caring for multiple patients in the same room could hinder infection prevention behaviours. An example was when a patient in the next bed asks for help, and they reported the dilemma of balancing infection prevention work with the desire to assist patients.

### Stress, high workload, and lack of time can lead to both conscious and unconscious deviations

Common reasons cited for inconsistent compliance with hygiene routines were stress, high workload, and lack of time. *If you feel like you have a lot to do and you don’t have enough time. You don’t want to hand it off to the next colleague, because you feel bad about it. So, you do things as quickly and efficiently as you can, maybe at the expense of hygiene routines, I would say* (RN3) (Interview 5). Participants noted various stressors, including high-need patients, emergencies, the need to double-check colleagues, staffing shortages, and personal concerns. Allocating resources and ability to care for fewer patients reduced stress. Overall, workload was perceived as high, while stress levels fluctuated throughout shifts and seasonally for some units. Some described an underlying feeling that their workload could intensify at any time, even during less stressful periods. The participants also mentioned that stress may be perceived and handled differently by different people and that some stress can be positive. Nurses who recently graduated found it difficult and stressful to set priorities.

Participants described how a lack of time and stress could lead to hygiene routines being overlooked, incomplete, or skipped altogether to save time. They emphasised the significance of having time for preparations, planning, reflection, and coordination. However, these activities could be deprioritised under stress, resulting in decreased compliance. Failures in preparation could cause staff members to forget necessary materials and make questionable decisions.

## Discussion

Infection prevention behaviours are influenced by the dynamic nature of the work environment at hospital units, particularly the multifaceted role of nursing staff. Nurses’ roles and work duties are often complex, and nursing staff are constantly faced with various dilemmas that they need to manage. Still, addressing individual working conditions may not ensure full compliance with infection prevention behaviours. Certain conditions are crucial and easily adaptable. For instance, the nursing staff reported that access to materials is essential, consistent with prior research (Zingg et al., 2015). However, situations can occur where infection prevention behaviours fail despite adequate physical conditions. Interruptions were emphasised as a risk for deviations in hygiene routines, and registered nurses were highlighted as particularly exposed, even if they accepted interruptions as an integral part of the nursing work. The phenomenon of interruptions in nursing work is well studied, and as in a previous study (Arvidsson et al., 2021), the participants stated that interruptions are common and that there is a lack of respect and understanding of when it is appropriate to interrupt a colleague. Managing interruptions can be easier for nurses with extensive experience (Laustsen and Brahe, 2018). Based on this, we can assume that it is easier for more experienced nurses to understand when it is suitable to interrupt colleagues and act appropriately when interrupted.

The nursing staff described how infection prevention could be deprioritised in acute or stressful situations, which is in line with studies showing that patient stability is prioritised over infection prevention (Weaver et al., 2023). However, deviations in infection prevention behaviours are not confined to emergencies. The importance of the climate and psychosocial work environment was highlighted, alongside challenges relating to staff turnover. In healthcare, staff turnover is common (Goodare, 2017), and globally, healthcare face ongoing strain and the need for continuous adaption (International Labour Organization, 2017). These are the working conditions under which nursing staff and their managers need to operate. Nursing staff hold primary responsibility for complying with hygiene routines. However, evidence from previous research indicates that both organisational factors and individual characteristics of healthcare professionals, such as knowledge, attitudes, and motivation, also play a significant role (Erasmus et al., 2010), further increasing the complexity.

In terms of compliance to hygiene routines, the nursing staff emphasised the importance of truly understanding infection prevention, which aligns with Sandberg’s theory (2000) that actions are based on one’s understanding of work and context (Sandberg, 2000). Personal experiences and interactions with colleagues shape this understanding, leading to behavioural change. However, Sandberg’s theory is rooted in the industrial sector, where contexts are more stable than in healthcare, a setting marked by unpredictability and complexity. Human factors, which focus on optimising performance by aligning systems and processes with human capabilities and limitations, play a key role in patient safety, as even skilled professionals may make errors in complex environments. A systematic review showed that interventions targeting physical ergonomics, cognitive processes, and organisational structures positively influence both staff outcomes and patient safety (Mao et al., 2015). These findings align with our study, highlighting how human factors influence infection prevention behaviours. Physical ergonomics is reflected in the need for accessible materials and equipment, as participants noted that poor placement or shortages hinder compliance. Cognitive factors are evident in interruptions, which affect focus and may lead to deviations from hygiene routines. Organisational factors, such as the psychosocial work environment, workload, and leadership, were reported by participants to impact their infection prevention behaviours.

Based on both our results and previous research highlighting the influence of human factors (Mao et al., 2015), as well as a review underscoring the importance of team training and simulations in preventing HCAIs (Costar and Hall, 2020), and drawing upon Sandberg’s (2000) theory that understanding increases through experience and collaboration (Sandberg, 2000), we can assume that sustaining infection prevention behaviours requires addressing multiple interconnected elements. First-line managers are ultimately responsible for their staff’s working conditions and have the authority to implement directed adjustments. Therefore, we recommend that managers implement activities such as team training with collegial reflection and educational initiatives where staff can also learn from each other, to enhance understanding of infection prevention practices. Practising and discussing risk situations could not only support individual staff members but also help to ensure that colleagues are not placed in situations with a risk for organism transmission, ultimately contributing to safer patient care. However, to establish and maintain sustainable working conditions in healthcare, it is critical to consider the complexities of the work environment and ensure adequate resources and support. Organisational decisions must address issues such as workload, staff turnover, and other overarching work-related factors to ensure that the hospital environment becomes a sustainable and supportive workplace for nursing staff, one that encourages people to join and remain in the profession.

## Methodological considerations

Credibility was enhanced by the diversity of settings and participants (Graneheim et al., 2017). Units varied in location, speciality, and size, while participants differed in age, profession, and experience, although the predominance of female participants was a limitation. The lack of criteria for employment tenure may affect credibility. Although groups were initially planned to have 6–8 participants, they comprised 3–7 due to staff availability, but discussions remained open and varied, supporting transferability. Representative quotations and dialogues are presented for transferability. Dependability was ensured by consistent data collection by the same two authors (LA and BS) with expertise in infection prevention and focus group techniques, and by using a consistent interview guide (Graneheim et al., 2017). It should be noted that self-reported behaviours may be influenced by social desirability bias, with participants potentially emphasising external factors over intrinsic motivations.

## Conclusion

This study highlights the complexities faced by nursing staff in maintaining infection prevention behaviours within the dynamic hospital work environment. Nursing staff are professionally obliged to comply with hygiene routines, and infection prevention behaviours can be influenced by individual as well as organisational factors. Therefore, managers and decision-makers must take responsibility for improving both physical and psychosocial working conditions, which, in turn, can help sustain safe practices.
